# Initial treatment of febrile seizures by mobile emergency care units in a cohort of children in the Region of Southern Denmark

**DOI:** 10.1186/1757-7241-23-S1-A6

**Published:** 2015-07-16

**Authors:** Morten O Holm, Søren Mikkelsen, Søren B Jepsen

**Affiliations:** 1 Department of Anesthesiology and Intensive Care, Odense University Hospital, Odense, Denmark; 2 Mobile Emergency Care Unit, Department of Anesthesiology and Intensive Care, Odense University Hospital, Odense, Denmark

## Background

Witnessing one's child having febrile seizures is a shocking experience to parents who may fear that their child is going to die. Febrile seizures are thus perceived as a serious illness and, accordingly, a physician-manned Mobile Emergency Care Unit (MECU) is often dispatched to the scene together with an ordinary ambulance. The aim of the study was to quantify the occurrence of febrile seizures and to assess the extent of treatment administered by the MECU in the Region of Southern Denmark.

## Methods

Retrospective observational study of the MECU database. The study is based on all 5,486 MECU runs in the Region of Southern Denmark involving children less than 15 years of age during the years 2010 - 2013. The included patients in the study were all assigned the diagnosis febrile seizures (R56.0) by the physician on site.

## Results

A total of 1,219 patients (22.2%) were assigned the diagnosis febrile seizures by the physician on site. Mean age was 24.5 months (ranging from 0 to 151 months). None of the patients died nor needed CPR. Three patients (0.2%) were intubated on site, 74 patients (6.1%) received anticonvulsive therapy intravenously by the physician, 343 patients (28.1%) were admitted to the hospital with physician escort, and 4 treatments (0.3%) were considered lifesaving by the treating physician.

## Conclusions

Febrile seizures were the largest diagnosis group in patients under 15 years of age in the cohort. Advanced medical treatment including tracheal intubation requiring a physician was rare. Further studies are warranted to determine the need for MECU physician treatment of febrile seizures.

